# Therapeutic effects and mechanisms of *Artemisia* species on metabolic diseases: A systematic review

**DOI:** 10.1097/MD.0000000000049379

**Published:** 2026-06-19

**Authors:** Naiyu Wang, Minmin Li, Mingjing Lu, Zheng Wang, Hualin Wang, Meiyue Yao, Hailing Ding, Muhammad Shahbaz, Muhammad Ijaz

**Affiliations:** a The Faculty of Medicine, Qilu Institute of Technology, Jinan, China; b Department of Pharmacy, Sichuan Orthopedic Hospital, Chengdu, China; c Department of Radiology, Qilu Hospital of Shandong University, Jinan, China.

**Keywords:** *Artemisia*, diabetes, metabolic disease, obesity, pharmacology

## Abstract

**Background:**

The prevalence of metabolic disorders has increased significantly in recent years, driving interest in effective herbal remedies. *Artemisia* species, part of the Compositae family, encompass over 500 plants worldwide and have shown promising potential in addressing metabolic ailments. This review delineates the intricate chemical composition of *Artemisia* plants while offering a thorough summary of the pharmacological advancements and clinical evidence of *Artemisia* species in mitigating conditions such as diabetes, hyperlipidemia, nonalcoholic fatty liver disease, obesity, and gout.

**Methods:**

A systematic review was conducted following Preferred Reporting Items for Systematic Reviews and Meta-Analyses guidelines. PubMed, Google Scholar, and Science Direct were searched up to December 2025. Studies evaluating *Artemisia* species for metabolic diseases (diabetes, hyperlipidemia, nonalcoholic fatty liver disease, obesity, or gout) were included. The included studies were analyzed and compared in terms of research types (animal, human, and in vitro studies), therapeutic outcomes, and mechanistic pathways. A qualitative synthesis was performed.

**Results:**

Numerous experiments have demonstrated the efficacy of 28 *Artemisia* species in lowering blood sugar and lipid levels, stimulating insulin secretion, ameliorating insulin resistance, suppressing inflammation and oxidative stress, reducing fat synthesis, and modulating the gut microbiota. These findings underscore their potential as promising therapeutic candidates for the management of metabolic disorders.

**Conclusion:**

This review acts as a crucial guide for steering the progress of drug development and the therapeutic use of *Artemisia* plants in addressing metabolic diseases. However, clinical investigations into the effects of *Artemisia* on metabolic disorders in humans are still limited. Further trials are essential to validate its effectiveness in treating these ailments.

## 1. Introduction

As the living environment evolves and dietary quality improves, there has been a global increase in the prevalence of metabolic disorders, including diabetes, obesity, hyperlipidemia, nonalcoholic fatty liver disease (NAFLD), and gout, in recent decades.^[1]^ Diabetes manifests as a metabolic disorder characterized by persistent hyperglycemia and disruptions in the metabolic processes of carbohydrates, proteins, and lipids.^[2]^ In 2025, the global prevalence of diabetes among people aged 20 to 79 years was estimated to be 11.1%, encompassing approximately 589 million individuals.^[3]^ Obesity is a long-lasting and intricate ailment marked by extravagant deposition of adipose tissue, which can adversely affect health and diminish quality of life.^[4]^ Hyperlipidemia, marked by high levels of total cholesterol (TC), triglycerides (TG), and low-density lipoprotein (LDL) cholesterol in the circulation, is the predominant manifestation of dyslipidemia. This condition is widespread in numerous countries and is primarily attributed to shifts in lifestyle.^[5]^ According to 2025 data, NAFLD prevalence is continuing to increase globally, affecting approximately 38% of the world’s adult population and between 7% and 14% of children and adolescents. By 2040, the global prevalence of NAFLD is projected to reach more than 55%.^[6]^ Dysregulation of glucose and lipid metabolic pathways, driven by the global surge in the incidence of type 2 diabetes mellitus, is likely the primary catalyst for the rising prevalence of NAFLD.^[7]^ In addition, gout, a metabolic anomaly marked by the aggregation of monosodium urate (MSU) crystals within joints, tissues, and organs, has experienced a worldwide increase in occurrence. This increase can be attributed to a combination of factors, including the aging population and rising incidence of obesity.^[8]^

The increasing incidence of metabolic diseases is a cause for concern. Although a range of efficacious medications for treating metabolic disorders has been identified over time, the concomitant adverse effects are disheartening.^[9,10]^ For example, metformin may induce hyperlactatemia and metabolic acidosis.^[11]^ In response, researchers have increasingly focused on botanical medicine. The utilization of medicinal plants that exhibit antidiabetic properties has been formally acknowledged in numerous countries.^[12]^ *Artemisia* is one of the most substantial and widely dispersed genera within the *Asteraceae* family, comprising over 500 species, and is primarily situated within temperate regions spanning Europe, North America, and Asia.^[13]^ These plants are widely used to treat bronchitis, malaria, epilepsy, irregular menstrual cycles, morning sickness, typhoid, and renal problems.^[14]^ In recent years, many studies have documented the hypoglycemic, hypolipidemic, and antioxidant properties of *Artemisia* plants.^[15,16]^ Hence, they are regarded as important natural remedies for the management of metabolic disorders. In this review, we present an extensive compilation of pharmacological evidence on the use of *Artemisia* spp. in addressing diabetes, obesity, hyperlipidemia, NAFLD, and gout. This serves as an indispensable resource for future research and development related to the genus *Artemisia*.

## 2. Methods

### 2.1. Search strategy

This study involved a systematic computerized search of PubMed, Google Scholar, and Science Direct to identify relevant studies published up to December 2025. Search terms were selected to maximize search sensitivity and specificity. The search strategy combined terms related to metabolic diseases, underlying mechanisms, and the plant genus *Artemisia* using Boolean operators (AND/OR). The full set of keywords used in the database searches included the following: terms related to metabolic diseases/disorders (metabolic diseases, diabetes, diabetes mellitus, obesity, hyperlipidemia, NAFLD, and gout); terms related to underlying mechanisms (hypoglycemic effects, insulin secretion, insulin resistance, enzymes, inflammation, oxidative stress, adipogenesis, and gut microbiota); terms related to the plant genus (*Artemisia*); and additional terms used in combination (pharmacology, clinical, and constituent*). The following search syntax illustrates the core search strategy employed in PubMed: (Metabolic diseases OR Diabetes Mellitus OR Hyperlipidemia OR Non-alcoholic Fatty Liver Disease OR Obesity OR Gout OR Constituent* OR Pharmacology OR Clinical OR hypoglycemic effects OR insulin secretion OR insulin resistance OR enzymes OR inflammation OR oxidative stress OR adipogenesis OR gut microbiota) AND (*Artemisia*). Similar search strategies were adapted for use in Google Scholar and Science Direct. All search results were exported to EndNote X9 for duplication removal. The complete search strategy has been reported in accordance with Preferred Reporting Items for Systematic Reviews and Meta-Analyses 2020 guidelines.

### 2.2. Eligibility criteria

A set of predefined criteria was employed to evaluate the eligibility of studies for inclusion in this systematic review. Population: The study population included animal models or human subjects with at least one of the following metabolic diseases: diabetes mellitus, hyperlipidemia, NAFLD, obesity, or gout. In vitro mechanistic studies using cell lines or primary cells relevant to these metabolic conditions were also included. Intervention: The intervention involved any *Artemisia* species or its preparations, including crude extracts or powdered plant material. No restrictions were applied regarding dosage, route, or duration of administration. Comparator: Eligible studies were required to include a control group receiving placebo, no treatment, or a standard comparator. Study types: Eligible study designs included controlled in vivo animal studies, human clinical studies, and in vitro experimental studies. The following were excluded: conference abstracts, books, dissertations, studies without full text or with incomplete data, studies lacking a valid control group, and studies not published in English. Duplicate data were excluded from the analysis to ensure the robustness and reliability of the findings.

### 2.3. Data extraction

Two independent reviewers extracted data using a standardized form. Disagreements were resolved by discussion or a third reviewer. Extracted information included study characteristics (e.g., author, year, country, design, sample size); population (e.g., species, metabolic disease type, baseline parameters); intervention (e.g., *Artemisia* species, form, dosage, route, duration); comparator (e.g., placebo, vehicle, no treatment, or standard care); therapeutic outcomes (e.g., blood glucose levels, blood lipid levels, facilitating insulin secretion, mitigating insulin resistance, inhibiting pivotal enzymes, reducing inflammation and oxidative stress, inhibiting adipogenesis, gut microbiota, body weight); mechanistic outcomes (e.g., adenosine monophosphate-activated protein kinase [AMPK], nuclear factor κ-light-chain-enhancer of activated B cells [NF-κB], phosphatidylinositol 3-kinase [PI3K]/protein kinase B [Akt]); and adverse effects. For animal studies, SYRCLE’s Risk of Bias tool (SYRCLE, Radboud University Medical Center) was employed. For human clinical studies, the Cochrane RoB 2.0 tool (Cochrane Methods Bias Group, Cochrane Collaboration) was used. Data on comorbidities were not consistently reported and were excluded. A third reviewer verified the extracted data for accuracy. A qualitative narrative synthesis was performed. Studies were grouped by metabolic disease category. Meta-analysis was not conducted due to substantial heterogeneity across studies (differences in *Artemisia* species, extract types, dosages, models, and outcomes). Due to the absence of a pre-registered protocol or trial registration information for most included studies (primarily animal and in vitro studies), formal assessment of reporting bias was not feasible.

### 2.4. Ethics approval

As this study is a systematic review of previously published literature and does not involve direct human participation or primary data collection, ethical approval and informed consent were not required. The review was conducted and reported in accordance with the Preferred Reporting Items for Systematic Reviews and Meta-Analyses guidelines.

## 3. Phytochemistry of *Artemisia* plants

**Figure 1.**
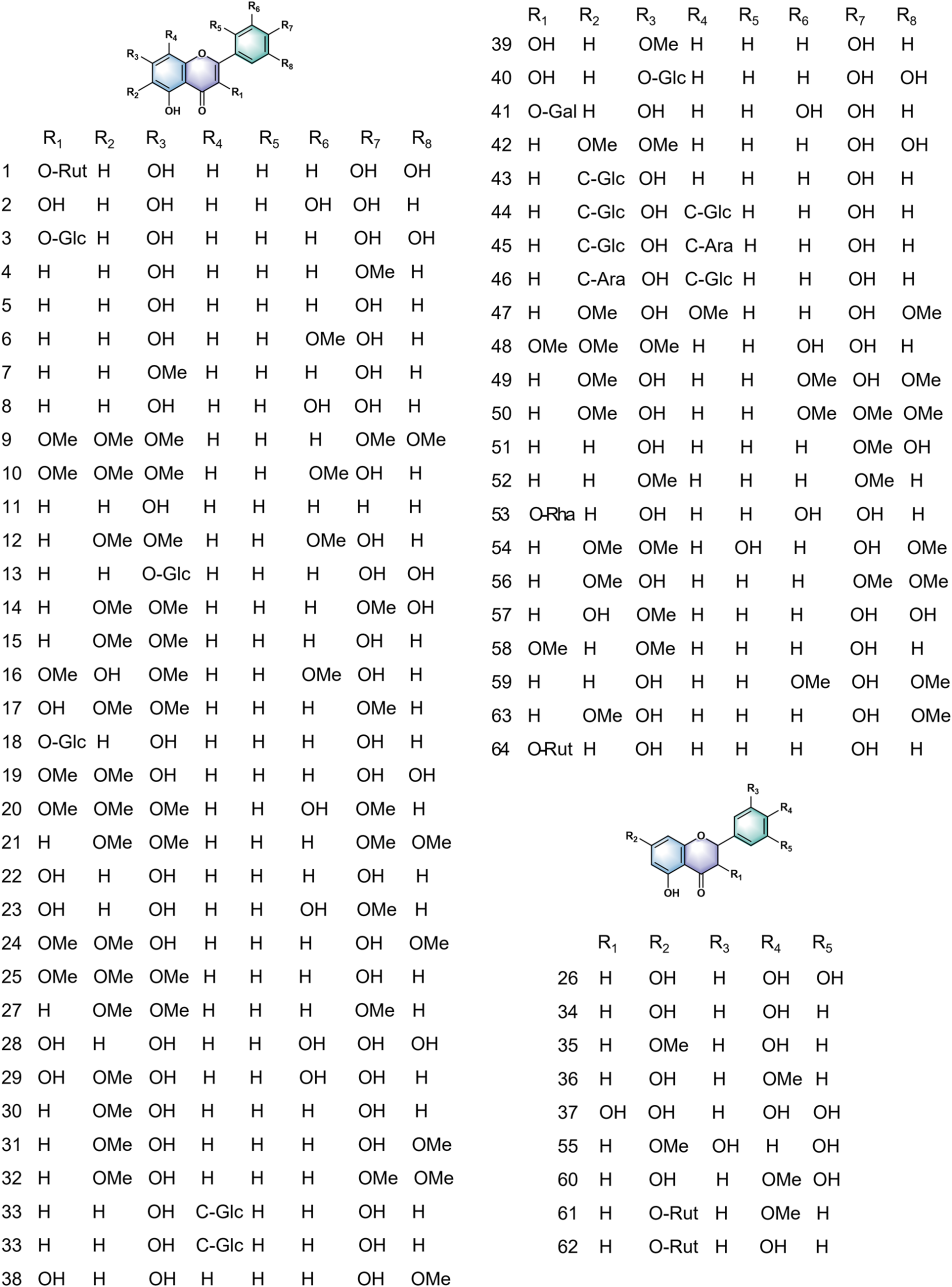
Chemical structures of flavonoids in *Artemisia.*

**Figure 2.**
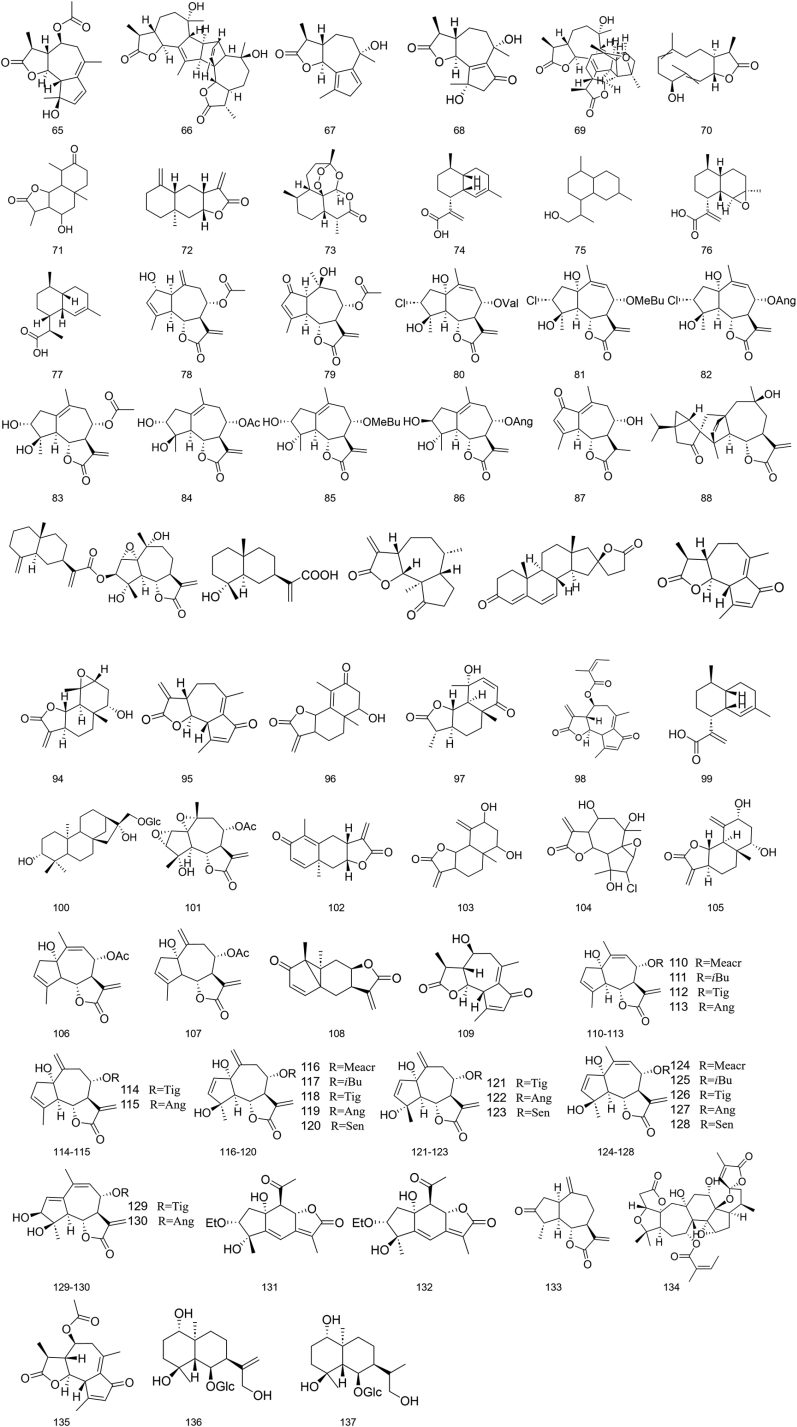
Chemical structures of terpenoids in *Artemisia.*

**Figure 3.**
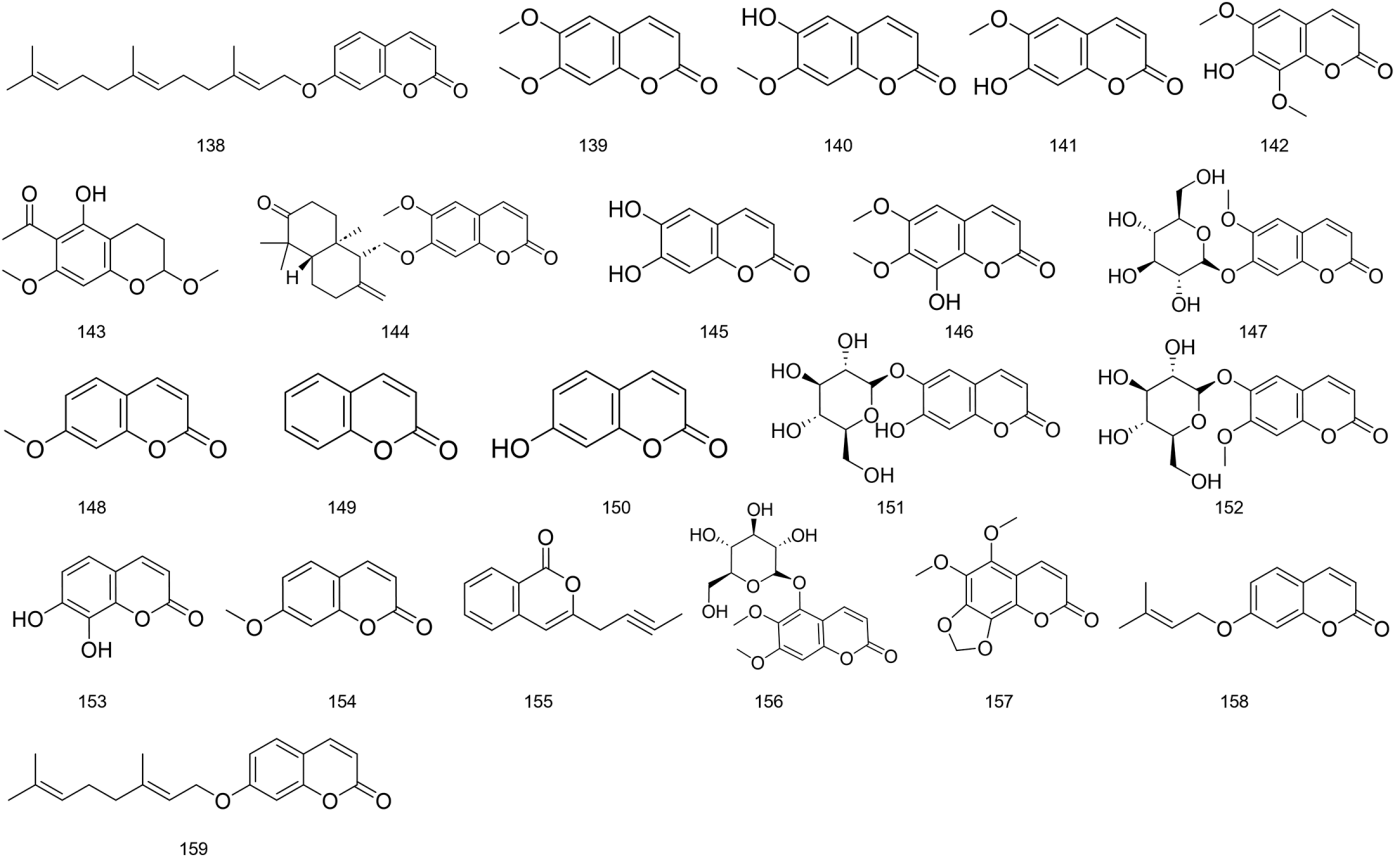
Chemical structures of coumarins in *Artemisia.*

**Figure 4.**
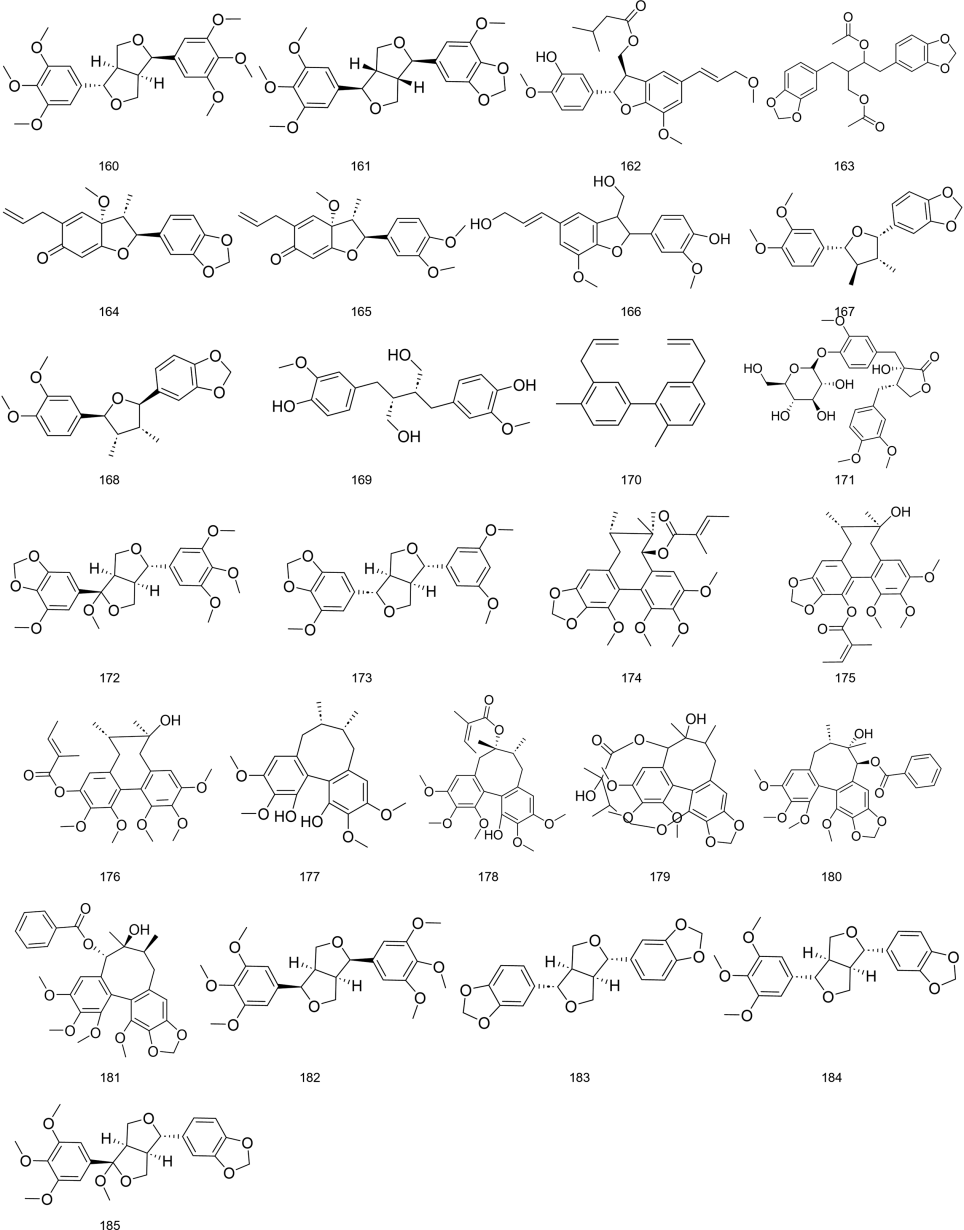
Chemical structures of lignans in *Artemisia.*

Recently, there has been widespread documentation regarding the isolation of phytochemical constituents from *Artemisia* spp. Flavonoids, terpenoids, coumarins, and lignans have been discerned as characteristic elements of *Artemisia* plants.^[13,17]^ Moreover, evidence indicates that they are pivotal bioactive metabolites chiefly accountable for the significance of these plants in medicine and pharmacy.^[18,19]^ Table 1^[20–95]^ presents the chemical constituents of *Artemisia*, with flavonoid compounds depicted in Figure 1, terpenoid compounds in Figure 2, coumarin compounds in Figure 3, and lignan compounds in Figure 4.

**Table 1:** Phytochemical constituents of diverse *Artemisia* species.

Plant source	Plant parts	Compound class and name	References
		Flavonoids	
*A absinthium* L.	–	Rutin (1), Quercetin (2), Isoquercitrin (3)	[20]
*A afra* Jacq	Aerial parts	Acacetin (4), Apigenin (5), Chrysoeriol (6), Genkwanin (7)	[21,22]
	Leaf	Luteolin (8)	[23]
*A annua* L	**–**	Rutin (1), Acacetin (4), Chrysoeriol (6), Artemetin (9), Chrysosplenetin (10), Chrysin (11), Cirsilineol (12), Cynaroside (13), Eupatorin (14), Cirsimaritin (15), Chrysosplenol-C (16), Mikanin (17), Astragalin (18), Axillarin (19), Casticin (20), Eupatin (21), Kaempferol (22), Tamarixetin (23), Jaceidin (24), Penduletin (25), Axillarin (19), Eriodictyol (26), Salvigenin (27), Myricetin (28)	[24–26]
	Aerial parts	Rutin (1), Quercetin (2), Isoquercitrin (3), Apigenin (5), Luteolin (8), Artemetin (9), Chrysosplenetin (10), Cirsilineol (12), Eupatorin (14), Casticin (20), Patuletin (29), Chrysosplenol-D (30)	[27–29]
*A argyi* Lévl.	Leaf	Quercetin (2), Apigenin (5), Luteolin (8), Kaempferol (22)	[30,31]
	–	Rutin (1), Quercetin (2), Apigenin (5), Luteolin (8), Hispidulin (30), Jaceosidin (31), Eupatilin (32), Vitexin (33)	[32,33]
*A campestris* L.	Aerial parts	Quercetin (2), Apigenin (5), Naringenin (34), Sakuranetin (35), Isosakuranetin (36)	[34,35]
	Whole plants	Rutin (1), Quercetin (2), Isoquercitrin (3), Apigenin (5), Luteolin (8), Cynaroside (13), Kaempferol (22), Naringenin (34), Taxifolin (37), Isorhamnetin (38)	[36]
*A capillaries* Thunb.	**–**	Apigenin (5), Genkwanin (7), Cynaroside (13), Cirsimaritin (15), Astragalin (18), Naringenin (34), Rhamnocitrin (39), Quercimeritrin (40), Hyperoside (41)	[37]
	Whole plants	Quercetin (2)	[38]
*A dracunculus* L.	**–**	Rutin (1), Quercetin (2), Luteolin (8), Kaempferol (22), Naringenin (34), Sakuranetin (35)	[39]
*A herba-alba* Asso.	Aerial parts	Acacetin (4), Chrysoeriol (6), Chrysin (11), Cirsilineol (12), Cirsimaritin (15), Astragalin (18), Casticin (20), Hispidulin (30), Jaceosidin (31), Eupatilin (32), Vitexin (33), Cirsiliol (42)	[40–43]
	Leaf and stems	Isovitexin (43), Vicenin-2 (44), Schaftoside (45), Isoschaftoside (46)	[44]
*A indica* Willd.	Leaf	Luteolin (8), Artemetin (9), Chrysosplenetin (10), Cirsilineol (12), Axillarin (19), Casticin (20), Eupatin (21), Sudachitin (47), Chrysophenol D (48),	[45,46]
	Aerial parts	Apigenin (5), Tamarixetin (23)	[22]
*A iwayomogi* Kitam.	Aerial parts	Rutin (1), Quercetin (2), Isoquercitrin (3), Apigenin (5), Genkwanin (7), Kaempferol (22), Patuletin (29), Hispidulin (30), Jaceosidin (31), Isorhamnetin (38), 6-methoxytricin (49), Arteanoflavone (50),	[47–49]
*A judaica* L.	Aerial parts	Apigenin (5), Luteolin (8), Cirsimaritin (15), Isovitexin (43), Diosmetin (51)	[50]
*A montana* Pamp.	Whole plants	Quercetin (2), Isoquercitrin (3), Apigenin (5), Luteolin (8), Hyperoside (41)	[51]
	Leaf	Apigenin (5), Chrysoeriol (6), Luteolin (8), Axillarin (19), Jaceosidin (31), Sudachitin (47)	[46]
*A princeps* Pamp.	**–**	Jaceosidin (31), Eupatilin (32)	[52,53]
*A roxburghiana* Wall.	Leaf and stems	Apigenin-7,4-dimethyl ether (52)	[54]
*A sacrorum* L.	Aerial parts	Quercetin (2), Acacetin (4), Apigenin (5), Genkwanin (7), Luteolin (8), Kaempferol (22), Hispidulin (30), Jaceosidin (31), Quercitrin (53)	[55]
*A scoparia* Waldst.	Aerial parts	Quercetin (2), Artemetin (9), Axillarin (19), Casticin (20), Eriodictyol (26), Naringenin (34), Sakuranetin (35), Isorhamnetin (38), Arcapillin (54), Blumeatin (55)	[56]
	Flower bud	Eupafolin (56), Pedalitin (57), Kumatakenin (58)	[57]
*A sieversiana* Willd.	Aerial parts	Rutin (1), Chrysoeriol (6), Chrysosplenetin (10), Tricin (59)	[58]
*A sphaerocephala* Krasch.	Whole plants	Eriodictyol (26), Naringenin (34), Isosakuranetin (36), Hesperetin (60), Didymin (61)	[59,60]
	Seeds	Quercetin (2), Apigenin (5), Taxifolin (37), Naringin (62)	[61]
*A vulgaris* L.	Aerial parts	Rutin (1), Quercetin (2), Apigenin (5), Kaempferol (22), Hyperoside (41), Quercitrin (53)	[29,34]
	Whole plants	Rutin (1), Isoquercitrin (3), Apigenin (5), Chrysoeriol (6), Luteolin (8), Astragalin (18), Vitexin (33), Hyperoside (41), Diosmetin (51), Quercitrin (53), Eupafolin (56), Jaceosidine (63), Nicotifloin (64)	[62]
		Terpenoids	
*A absinthium* L.	–	Matricin (65), Absinthin (66), Artabsin (67), Artemolin (68), Anabsinthin (69), Artabin (70), Arabsin (71)	[19]
*A afra* Jacq	Leaf	Isoalantolactone (72)	[63]
*A annua* L	–	Artemisinin (73), Artemisinic acid (74), Artemisinol (75), Epoxyartemisinic acid (76), Dihydroartemisic acid (77)	[64]
*A argyi* Lévl.	–	Argyinolide A-I (78–86), Deacetylmatricatin (87), Isoartemisolide (88), Artemilinin A (89), Ilicic acid (90)	[65]
*A campestris* L.	Aerial parts	Damsin (91), Canrenone (92)	[66]
*A capillaries* Thunb.	Aerial parts	Leucodin (93), Ludovicin A (94), Dehydroleucodin (95), Armexifolin (96)	[67]
*A judaica* L.	Aerial parts	Vulgarin (97)	[68]
*A herba-alba* Asso.	Aerial parts	Moxartenolide (98), Artemisinic acid (99)	[42]
*A iwayomogi* Kitam.	Aerial parts	Iwayoside A-B (100–101), Yomogin (102), Ridentin B (103), Bibsanin (104), Ludovicin B (105), Lupicolin A acetate (106), Lupicolin B acetate (107), Lumiyomogin (108)	[69]
*A princeps* Pamp.	Aerial parts	Desacetylmatricarin (109)	[70]
*A sacrorum* L.	Aerial parts	Artemisacrolides A–W (110–132)	[71]
*A scoparia* Waldst.	Aerial parts	Estafiatone (133), Ludovicin B (105)	[72]
*A sieversiana* Willd.	Aerial parts	Sieverlactone (134), Matricarin (135), Desacetylmatricarin (109)	[73]
*A vulgaris* L.	Aerial parts	Vulgarosides A-B (136–137)	[74]
		Coumarins	
*A absinthium* L.	–	Umbelliprenin (138)	[75]
*A afra* Jacq	Seeds	Scoparone (139), Isoscopoletin (140)	[76]
*A annua* L	Leaf and stems	Scoparone (139), Scopoletin (141), Isofraxidin (142)	[77]
	–	Scopoletin (141), Qinghaocoumarin A (143), Qinghaocoumarin B (144)	[78]
*A argyi* Lévl.	Leaf	Isoscopoletin (140), Scopoletin (141)	[79]
*A campestris* L.	–	Scopoletin (141), Esculetin (145), Fraxidin (146), Scopolin (147), Herniarin (148)	[80]
*A capillaries* Thunb.	Whole plants	Scoparone (139), Isoscopoletin (140), Scopoletin (141), Esculetin (145), Scopolin (147), Coumarin (149), Umbelliferone (150), Esculin (151), Isoscopolin (152), Daphnetin (153), 7-Methoxycoumarin (154)	[81]
*A dracunculus* L.	–	Scoparone (139), Scopoletin (141), Esculetin (145), Coumarin (149), Esculin (151), Capillarin (155)	[82]
*A herba-alba* Asso.	Aerial parts	Tomenin (156)	[83]
*A iwayomogi* L.	Whole plants	Scopoletin (141), Scopolin (147)	[49]
*A montana* Pamp.	Whole plants	Scoparone (139), Scopoletin (141), Esculetin (145), Scopolin (147), Umbelliferone (150)	[51]
*A princeps* Pamp.	Leaf	Scopoletin (141), 7-Methoxycoumarin (154)	[84,85]
*A sacrorum* L.	Aerial parts	Isoscopoletin (140), Scopoletin (141)	[86]
*A scoparia* Waldst.	Aerial parts	Scoparone (139), Scopoletin (141), Isofraxidin (142), Isosabandin (157)	[56]
*A vulgaris* L.	Aerial parts	Umbelliprenin (138), 7-Isopentenyloxycoumarin (158), Auraptene (159)	[87]
		Lignans	
*A absinthium* L.	Aerial parts	Epiyangambin (160), Sesartemin (161)	[88]
*A annua* L	Aerial parts	Qinghaolignan A (162), Qinghaolignan B (163), Denudatin A (164), Denudatin B (165), Dehydrodiconiferyl alcohol (166), Futokadsurin B (167), Futokadsurin C (168)	[78]
*A argyi* Lévl.	–	Secoisolariciresinol (169), Honokiol (170)	[89,90]
*A campestris* L.	Aerial parts	Tracheloside (171)	[91]
*A sieversiana* Willd.	Aerial parts	Sieverlignan A-E (172–176), Rubrisandrin B (177), Micrantherin A (178), Gomisin D (179), Gomisin G (180), Schisantherin A (181), Epiyangambin (160), Diyangambin (182), Sesamin (183), Ashantin (184), Carullignan B (185)	[92–95]

## 4. Pharmacological effects and mechanisms of *Artemisia* species on metabolic disorders

**Figure 5.**
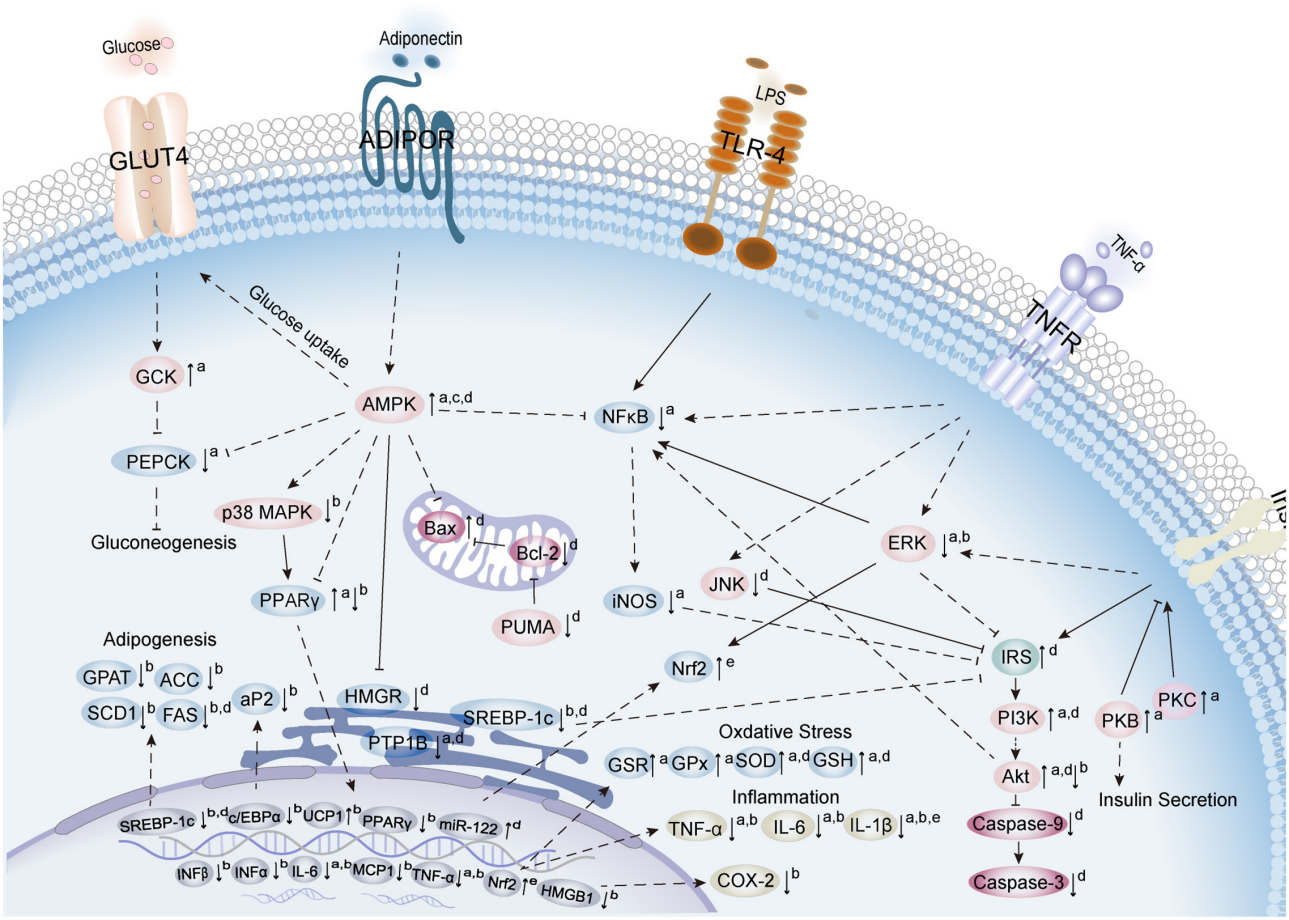
*Artemisia* species can ameliorate diabetes, hyperlipidemia, and NAFLD management by influencing key targets. ↑ indicates an increase, and ↓ indicates a decrease. → indicates a stimulatory effect, and ⊣ indicates an inhibitory effect. In the top right corner, a depicts the effect of *Artemisia* on diabetes, b indicates the effect of *Artemisia* on obesity, c demonstrates the effect of *Artemisia* on hyperlipidemia, d represents the effect of *Artemisia* on NAFLD, and e represents the effect of *Artemisia* on gout. Pathway information is from the KEGG database. ACC = acetyl-CoA carboxylase, ADIPOR = adiponectin receptor, Bax = BCL-2-associated X, Bcl-2 = B-cell lymphoma-2, C/EBPα = CCAAT/enhancer binding protein-α, COX-2 = hepatic cyclooxygenase-2, GLUT4 = glucose transporter type 4, GPAT = glycerol-3-phosphate acyltransferase, GPx = glutathione peroxidase, GSR = glutathione reductase, IL-6 = interleukin-6, iNOS = inducible nitric oxide synthase, NAFLD = nonalcoholic fatty liver disease, PEPCK = phosphoenolpyruvate carboxykinase, PI3K = phosphatidylinositol 3-kinase, PKB = protein kinase B, PKC = protein kinase C, PPARγ = peroxisome proliferator-activated receptor γ, TLR-4 = toll-like receptor 4, TNF-α = tumor necrosis factor-α, TNF-R = tumor necrosis factor receptor superfamily member 1A, UCP1 = uncoupling protein 1.

*Artemisia* species have been extensively used in traditional medicine for the management of metabolic disorders. This review provides a comprehensive synthesis of the pharmacological activities and mechanisms underlying the utilization of *Artemisia* species in the management of diabetes, obesity, hyperlipidemia, NAFLD, and gout. The administration details of *Artemisia* species in the included in vivo studies are shown in Table 2,^[96–146]^ and the principal targets modulated by *Artemisia* species are shown in Figure 5.

**Table 2:** Administration details of *Artemisia* species in the included in vivo studies.

*Artemisia* species	Amount	Form	Route of administration	Duration of administration	References
Hypoglycemic effect of *Artemisia* species					
* A. herba-alba*	0.39 g/kg body weight	Aqueous extract	Oral administration	2–4 wk	[96]
* A. herba-alba*	0.39 g/kg body weight	Aqueous extract	Oral administration (intragastrically via cannula, 2 mL/kg)	Not reported	[97]
* A santonicum*	0.42 g/kg	Aqueous extract	Oral (by gavage, 2 mL/kg)	Not reported	[98]
* A dracunculus*	500 mg/kg body weight/d	Ethanolic extract	Gavage	7 d	[99]
* A afra*	50, 100, and 200 mg/kg	Aqueous leaf extract	Not reported	15 d	[100]
* A indica*	200 and 400 mg/kg	Hydromethanolic crude extracts	Oral administration	15 d	[101]
*Artemisia* species promote insulin secretion					
* A afra*	50 and 100 mg/kg	Aqueous leaf extract	Oral administration	14 d	[102]
* A herba-alba*	20 mg/rat	70% ethyl alcohol extract	Intraperitoneal injection	21 d	[103]
*Artemisia* species alleviate insulin resistance					
* A scoparia*	400 mg/d	Pharmaceutical-grade *A scoparia* extract (tablets, 99.9% purity)	Oral administration (consumption, 1 tablet after breakfast and 1 after dinner)	10 wk	[104]
* A scoparia*	1% w/w	Ethanolic extract	Oral administration (feeding, via high-fat diet containing SCO)	4 wk	[105]
* A sphaerocephala*	0.3%, 0.9%, 2.7% (w/w, in high-fat diet)	Seed powder obtained by water extraction and ethanol precipitation	Oral administration (mixed with food supply, added to high-fat diet)	8 wk	[106]
*Artemisia* species promote glucose uptake and glycolysis, and inhibit gluconeogenesis					
* A sphaerocephala*	0.3%, 0.9%, 2.7% (w/w, in high-fat diet)	Seed powder obtained by water extraction and ethanol precipitation	Oral administration (mixed with food supply, added to high-fat diet)	8 wk	[106]
* A sphaerocephala*	800 mg/kg	Polysaccharide fractions	Intragastric administration (oral gavage)	8 wk	[107]
*Artemisia* species inhibit inflammation					
* A judaica*	300 mg/kg	70% ethanol extract	Oral administration	28 d	[108]
*Artemisia* species inhibit several key enzymes					
* A ludoviciana*	31.6, 100, and 316 mg/kg	Essential oil	Oral administration	Not reported	[109]
*Artemisia* species inhibit oxidative stress in diabetic animals					
* A turanica*	70 mg/kg	70% ethanol extract	Oral administration	4 consecutive weeks	[110]
* A herba-alba*	400 mg/kg	Aqueous extract	Oral administration	30 d	[111]
* A campestris*	200 mg/kg	Aqueous extract	Intraperitoneal injection	21 d	[112]
* A afra*	50 and 100 mg/kg	Aqueous extract	Oral administration	21 d	[113]
*Artemisia* species inhibits adipogenesis and prevents obesity					
* A annua*	150 mg/kg	80% ethanol extract	Oral administration	5 wk	[114]
* A annua*	200 mg/kg	Aqueous extract	Oral administration	7 wk	[115]
*Artemisia* species reduces inflammation in HFD-fed mice					
* A sphaerocephala*	400 mg/kg/d	Polysaccharide	Intragastric administration (oral gavage)	8 wk	[116]
* A annua*	400 mg/kg	80% aqueous ethanol extract	Dietary administration (mixed with diet)	12 wk	[117]
* A iwayomogi*	0.5% (w/w)	Ethanol extract	Oral administration (dietary administration, mixed with diet)	11 wk	[118]
*Artemisia* species alleviates gut microbiota dysbiosis in animals with obesity					
* A sphaerocephala*	200 mg/kg	Polysaccharide	Intragastric administration (oral gavage)	12 wk	[119]
* A sphaerocephala*	200, 400, and 800 mg/kg/d	Polysaccharide	Intragastric administration (oral gavage)	8 wk	[120]
* A sphaerocephala*	400 mg/kg/d	Polysaccharide	Intragastric administration (oral gavage)	8 wk	[116]
Hyperlipidemia					
* A capillaris*	50 and 100 mg/kg	Aqueous extract	Oral administration	7 d	[121]
* A integrifolia*	100 and 200 mg/kg	Petroleum ether extract	Oral administration	15 d	[122]
* A iwayomogi*	100 mg/kg	30% ethanol extracts	Oral administration	10 wk	[123]
* A sphaerocephala*	0.10, 0.15, and 0.20 mL/10 g/d	Seed oil	Oral administration	30 d	[124]
* A vulgaris*	25 and 50 mg/kg	Aqueous extract	Oral administration (by oral feeding needle with tuberculin syringe)	30 d	[125]
* A iwayomogi*	0.1%, 0.25%, and 0.5% w/w	50% ethanol extract	Oral administration (mixed with diet)	10 wk	[126]
NAFLD					
* A annua*	50 and 100 mg/kg	Aqueous extract	Intragastric administration (oral gavage)	8 wk	[127]
* A capillaris*	0.462, 0.924, and 1.848 g/kg body weight/d	ACF powder was dissolved in physiological saline to a working concentration of 500 mg/mL	Intragastric administration	4 wk	[128]
* A capillaris*	50 mg/kg/d	Aqueous extract	Intragastric administration (gavage twice a week)	6 wk	[129]
* A scoparia*	250 mg/kg	80% ethanol extract	Oral administration (mixed with diet)	6 wk	[130]

ACF = aberrant crypt foci, HFD = high-fat diet, NAFLD = nonalcoholic fatty liver disease, SCO = scopoletin.

### 4.1. Diabetes

Numerous studies have demonstrated the efficacy of *Artemisia* species in the management of diabetes. The mechanisms underlying these effects include reducing blood glucose levels, facilitating insulin secretion, mitigating insulin resistance, and inhibiting pivotal enzymes, inflammation, and oxidative stress.

#### 4.1.1. Hypoglycemic effect of *Artemisia* species

Certain species of *Artemisia* have demonstrated hypoglycemic effects in animal models of diabetes. Multiple studies have indicated that administration of an aqueous extract of *Artemisia herba-alba* (390 g/kg) notably lowered blood glucose levels.^[96,97]^ Similarly, the oral application of an aqueous-based extract of *Artemisia santonicum* (0.42 g/kg, i.g.) led to a notable reduction in blood glucose levels in diabetic rabbits.^[98]^ Ribnicky et al noted that in genetically diabetic Kuo Kondo-Aγ mice, the administration of *Artemisia dracunculus* ethanolic extract (500 mg/kg, i.g., for 7 days) resulted in a 24% decline in blood sugar levels.^[99]^ Furthermore, administration of *Artemisia afra* aqueous extracts at doses of 50, 100, and 200 mg/kg for 15 days led to a reduction in blood sugar levels and enhancement of glucose tolerance. At the highest dosage, the extracts exhibited the most potent hypoglycemic effect, equivalent to glibenclamide, a conventional antidiabetic medication.^[100]^ Moreover, compared with glibenclamide, the utilization of hydromethanolic and chloroform extracts derived from *Artemisia indica* (200 and 400 mg/kg, p.o., for 15 days) resulted in a marked decline in blood glucose levels.^[101]^

#### 4.1.2. *Artemisia* species promote insulin secretion

Several studies have demonstrated that *Artemisia* plants can enhance insulin secretion via various mechanisms. Through in vitro experimentation with medicinal plant extracts sourced from the Islamabad and Murree regions, Hussain et al discovered insulin secretagogue properties in the ethanol extract of *Artemisia roxburghiana* leaves when examined in INS-1 cells (an insulinoma cell line).^[131]^ The aqueous extract from *Artemisia capillaris* reinstated the suppression of insulin release induced by cytokines in isolated islets by suppressing NF-κB activity.^[132]^ PMI-5011, an alcoholic extract derived from *A dracunculus*, induces insulin secretion from primary β cells by activating AMPK and protein kinase B.^[133]^ Similarly, aqueous extracts of *A afra* (50 and 100 mg/kg, p.o., for 14 days) and 70% ethyl alcohol extract of *A herba-alba* (20 mg/rat, i.p., for 21 days) have the capacity to induce insulin release by regenerating β-cells in diabetic rats.^[102,103]^

#### 4.1.3. *Artemisia* species alleviate insulin resistance

Clinical research indicates that, in comparison to the placebo cohort, daily ingestion of pharmaceutical-grade *Artemisia scoparia* extract at a dose of 400 mg/day notably decreased the homeostasis model assessment of insulin resistance (HOMA-IR) in gestational diabetes following a 10-week treatment period.^[104]^ Similarly, mice treated with *A scoparia* ethanolic extract (1% w/w, p.o., for 4 weeks) exhibited markedly reduced insulin resistance, as demonstrated by quantitative assessment using the HOMA-IR method.^[105]^ Subsequently, research has suggested that treatment with PMI-5011 facilitates the activation of AKT serine/threonine kinase 1 and AKT serine/threonine kinase 2 through phosphorylation, concurrently decreasing the abundance of phosphatase-1B (PTP-1B) protein, thereby ameliorating insulin resistance.^[134,135]^ Furthermore, the seed powder of *Artemisia sphaerocephala* (0.3%, 0.9%, and 2.7% w/w, p.o., for 8 weeks) exhibits the capacity to mitigate insulin resistance in streptozotocin-induced type 2 diabetes mellitus rats by enhancing liver glucokinase activity, augmenting liver glycogen levels, and diminishing hepatic fat accumulation.^[106]^

#### 4.1.4. *Artemisia* species promote glucose uptake and glycolysis, and inhibit gluconeogenesis

Research indicates that *Artemisia* species can enhance glucose uptake and glycolysis while inhibiting gluconeogenesis. For example, PMI-5011 optimizes the organization of actin filaments, facilitating the translocation of glucose transporter-4 to the cellular membrane. This augmentation leads to enhanced glucose uptake, transit, and metabolic processes.^[136]^ Additionally, *Artemisia princeps* ethanolic extract enhances glucose uptake through a combination of the PI3K-dependent (phosphoinositide 3-kinase-dependent) atypical protein kinase C and AMPK pathways.^[137]^ An investigation demonstrated that the extract derived from the seed powder of *A sphaerocephala* (0.3%, 0.9%, and 2.7% w/w, p.o., for 8 weeks) diminished endogenous glucose production by facilitating the conversion of blood glucose into liver glycogen.^[106]^ Li et al found that *A sphaerocephala* polysaccharides (800 mg/kg, i.g., for 8 weeks) enhance glycogen synthesis by increasing the expression levels of glucose transporter-4, peroxisome proliferator-activated receptor-γ (PPARγ), PI3K, protein kinase B, and glucokinase, while inhibiting gluconeogenesis through the downregulation of phosphoenolpyruvate carboxykinase expression.^[107]^

#### 4.1.5. *Artemisia* species inhibit inflammation

*Artemisia* species have been shown to possess significant anti-inflammatory properties. Studies have indicated that treatment with PMI-5011 attenuated the cytokine-triggered initiation of inflammatory signaling cascades, including Erk1/2 and IkBα (nuclear factor of kappa light polypeptide gene enhancer in B-cells inhibitor)-NF-κB, along with the activation of NF-κB-regulated genes.^[138,139]^ Consistently, Aggarwal et al discovered that PMI-5011 effectively suppressed inflammation induced by lipopolysaccharide (LPS)/interferon-γ and restrained the generation of nitric oxide (NO) and inducible NO synthase while also mitigating the production of the pro-inflammatory cytokine interleukin-6 (IL-6).^[133]^ Moreover, administration of *Artemisia judaica* 70% ethanol extract to diabetic rats mitigated inflammation by reducing the level of tumor necrosis factor-α (TNF-α).^[108]^

#### 4.1.6. *Artemisia* species inhibit several key enzymes

It is widely recognized that α-glucosidase, protein tyrosine PTP-1B, dipeptidyl peptidase IV, and other inhibitors are promising therapeutic agents for diabetes treatment.^[140]^ In addition, methanol extracts from 12 *Artemisia* species, including *Artemisia apiacea*, *Artemisia rubripes*, *Artemisia japonica*, *Artemisia sylvatica*, *Artemisia iwayomogi*, *Artemisia argyi*, *A princeps*, *A capillaris*, *Artemisia annua*, *Artemisia sieversiana*, *Artemisia montana*, and *Artemisia stolonifera* have demonstrated concentration-dependent inhibitory activity against both α-glucosidase and PTP-1B.^[141]^ Moreover, Anaya-Eugenio et al discovered that an essential oil of *Artemisia ludoviciana* (31.6, 100, and 316 mg/kg, p.o.) resulted in 45% inhibition in an enzymatic test using yeast α-glucosidase.^[109]^ A hydromethanolic extract of *A judaica* exhibited inhibitory potential against α-glucosidase, α-amylase, and dipeptidyl peptidase IV, with half-maximal inhibitory concentration (IC50) values spanning from 758.96 to 2447.40 µg/mL.^[142]^ Following this, the entire plant of *A montana* demonstrated significant in vitro rat lens aldose reductase suppressing activity, with an IC50 value of 0.51 ± 0.06 μg/mL.^[51]^ Additionally, an ethanolic extract derived from *A dracunculus* attenuates the expression of phosphoenolpyruvate carboxykinase in rats with diabetes.^[143]^

#### 4.1.7. *Artemisia* species inhibit oxidative stress in diabetic animals

*Artemisia* species have demonstrated remarkable efficacy in mitigating oxidative stress. Research indicates that 70% ethanol extracts from *Artemisia turanica* (70 mg/kg, p.o., for 4 weeks) lead to elevated concentrations of superoxide dismutase (SOD) and diminished levels of malondialdehyde (MDA).^[110]^ Sekiou et al discovered that aqueous extracts of *A herba-alba* (400 mg/kg, p.o., for 30 days) enhanced antioxidant enzymatic activity and restored the levels of MDA and glutathione (GSH) to normalcy.^[111]^ Sefi et al found that the administration of aqueous extracts of *Artemisia campestris* (200 mg/kg, i.p., for 21 days) to diabetic rats caused a notable decrease in pancreatic levels of MDA, protein carbonyl content, and advanced oxidation protein products by 54%, 53%, and 20%, respectively.^[112]^ Aqueous extracts of *A afra* (50 and 100 mg/kg, p.o., for 21 days) elevated the levels of glutathione reductase, glutathione peroxidase, SOD, and GSH, restoring them to nearly normal levels.^[113]^

### 4.2. Obesity

Several studies have elucidated the advantages of *Artemisia* species in combating obesity. In essence, the anti-obesity efficacy of these species may arise from the inhibition of adipogenesis, mitigation of inflammation in adipose tissue, and regulation of dysbiosis in gut microbiota.

#### 4.2.1. *Artemisia* species inhibit adipogenesis and prevent obesity

Research indicates that administration of 80% ethanol extracts of *A annua* (150 mg/kg, p.o., for 5 weeks) notably reduced body weight gain, adipocyte cell size, adipose tissue mass, TC, and serum TG levels through the downregulation of major adipogenic transcription factors, C/EBPα (CCAAT/enhancer binding protein-α) and PPARγ, as well as Akt signaling, in obese rats.^[114]^ Water extracts of *A annua* (200 mg/kg, p.o., for 7 weeks) diminished the expression levels of PPARγ and C/EBPα in addition to the enzymes governing fatty acid metabolism. This reduction significantly attenuated adipocyte differentiation and white fat accumulation in the interscapular region. Concurrently, augmenting uncoupling protein 1 expression represses genes that foster adipocyte differentiation.^[115]^ Likewise, the expression of obesity-related proteins including PPARγ, C/EBPα, SREBP1c (sterol regulatory element-binding protein 1c), fatty acid synthase (FAS), and acetyl-CoA carboxylase, exhibited augmentation in 3T3-LI cells, which was subsequently mitigated upon treatment with *A annua* essential oil.^[144]^ Fifty percent ethanol extract of *Artemisia sacrorum* exerts downregulatory effects on the gene expression of SREBP1c and its downstream targets, including FAS, stearoyl-CoA desaturase 1, and glycerol-3-phosphate acyltransferase, as well as on PPARγ and C/EBPα. Moreover, the downstream target, adipocyte fatty acid-binding protein, was diminished in a concentration-dependent manner.^[145]^ In addition, administration of *A princeps* crude extract led to the downregulation of MAPK pathway protein activation, particularly p38 MAP kinase and extracellular signal-regulated kinase, subsequently reducing the expression of PPARγ, C/EBPα, and sterol regulatory element-binding protein-1c. This inhibition impedes adipocyte traits in differentiating 3T3-L1 pre-adipocytes, resulting in diminished adipogenesis and reduced intracellular lipid levels.^[146]^

#### 4.2.2. *Artemisia* species reduce inflammation in high-fat diet-fed mice

A high-fat diet can provoke inflammation, and certain species of *Artemisia* have demonstrated a notable alleviating effect. Upon administration of *A sphaerocephala* polysaccharide (400 mg/kg, i.g., for 8 weeks), serum concentrations of LPS, TNF-α, interleukin-1 beta, and IL-6 were significantly reduced, concurrently mitigating the marked inhibition of ZO-1 and occludin levels in high-fat diet-fed mice.^[116]^ After 12 weeks, administration of 80% ethanol extract of *A annua* (400 mg/kg, p.o.) lowered the expression of high-mobility group box 1 and led to a noteworthy reduction in hepatic cyclooxygenase-2 levels.^[117]^ The ethanol extract of *A iwayomogi* (0.5% w/w, p.o., for 11 weeks) notably downregulated pro-inflammatory cytokine genes (TNFα, INFβ, IL-6, and monocyte chemoattractant protein-1 and interferon alpha) in epididymal adipose tissue and lowered plasma concentrations of TNFα and monocyte chemoattractant protein-1 in comparison to the high-fat diet alone.^[118]^

#### 4.2.3. *Artemisia* species alleviate gut microbiota dysbiosis in animals with obesity

Research has shown that *A sphaerocephala* has the potential to ameliorate dysbiosis in the gut microbiota and to repair the integrity of the gut barrier in obese animals. Analysis through 16S rRNA gene sequencing revealed that *A sphaerocephala* polysaccharide (200 mg/kg, i.g., for 12 weeks) alleviated the disruption in gut microbiota caused by a high-fat diet and promoted the proliferation of beneficial probiotics, including Lactobacillus, Bifidobacterium, and Blautia.^[119]^ Similarly, 16S rRNA analysis revealed the capacity of *A sphaerocephala* polysaccharides (200, 400, and 800 mg/kg, i.g., for 8 weeks) to mitigate gut dysbiosis triggered by a high-fat diet, reducing Proteobacteria, Helicobacter, and AF12.^[120]^ In addition, research has shown that *A sphaerocephala* polysaccharides (400 mg/kg, i.g., for 8 weeks) mitigate gut dysbiosis in obese mice, notably augmenting Bifidobacterium and Olsenella (beneficial genera) while inhibiting Mucispirillum and Helicobacter (detrimental genera).^[116]^

### 4.3. Hyperlipidemia

Through an extensive array of experimental studies, the efficacy of *Artemisia* spp. in managing hyperlipidemia has been substantiated. Aqueous extracts of *A capillaris* (50 and 100 mg/kg, p.o., for 7 days) mitigated the increase in serum LDL cholesterol, TC, and TG levels.^[121]^ Studies have demonstrated that *A sphaerocephala* seed oil (0.10, 0.15, and 0.20 mL/10 g, i.g., for 30 days), as well as petroleum ether extracts from *Artemisia integrifolia* (100 and 200 mg/kg, i.g., for 15 days) and 30% ethanol extracts of *A iwayomogi* (100 mg/kg, p.o., for 10 weeks), markedly diminished the concentrations of TC and TG in hyperlipidemic animals.^[122–124]^ An aqueous extract derived from *Artemisia vulgaris* (25 and 50 mg/kg, p.o., for 30 days) has demonstrated remarkable efficacy in hyperlipidemic rats, leading to reductions in TC, TG, LDL, and very-low-density lipoprotein levels, along with an elevation in high-density lipoprotein (HDL) levels.^[125]^ Fifty percent ethanol extracts of *A iwayomogi* (0.1%, 0.25%, and 0.5% w/w, p.o., for 10 weeks) led to dose-dependent improvements in key indicators of hypertriglyceridemia, such as TG, free fatty acid (FFA), apolipoprotein B, and lipoprotein lipase, via the activation of AMPK-mediated signaling pathways. This activation downregulates molecules associated with lipogenesis while concurrently upregulating those involved in fatty acid oxidation.^[126]^

### 4.4. NAFLD

Studies have revealed that *Artemisia* contributes to the management of NAFLD via various mechanisms. An examination employing an aqueous extract of *A annua* (50 and 100 mg/kg, i.g., for 8 weeks) hindered lipid accumulation and shielded cells from oxidative stress-induced damage by activating antioxidant enzymes, such as SOD, while also enhancing the production of GSH during the treatment of NAFLD.^[127]^ As revealed by Liu et al, treatments involving *A capillaris* (0.462, 0.924, and 1.848 g/kg, i.g., for 4 weeks) significantly reduced the levels of TC and TG, suppressed the expression of FAS, and elevated microRNA 122 levels. These interventions effectively relieve diet-induced fatty liver disease and intracellular lipid accumulation stemming from fatty acids.^[128]^ In connection with this, aqueous extracts derived from *A capillaris* (50 mg/kg, i.g. twice weekly, for 6 weeks) stimulated the phosphorylation of AMPK and PI3K/AKT in human hepatocellular carcinoma cell line and liver cells of NAFLD animals. These extracts suppressed sterol regulatory element-binding protein-1c expression, diminished TG and lipogenesis, and attenuated lipid accumulation.^[129]^ Wang et al discovered that 80% ethanol extracts derived from *A scoparia* (250 mg/kg, p.o., for 6 weeks) reduced the abundance of SREBP1c, FAS, HMG-CoA reductase, and PTP-1B. Furthermore, these extracts notably increased hepatic insulin receptor substrate-2 content and phosphorylation levels of AKT serine/threonine kinase 1, AKT serine/threonine kinase 2, insulin receptor beta, and insulin receptor substrate-1, along with enhanced AMPK and AMP-activated protein kinase alpha 1 activity, thereby alleviating NAFLD.^[130]^ Thirty percent ethanol extract of *A capillaris* notably ameliorated FFA-induced steatosis and regulated BCL-2-associated X, B-cell lymphoma-2, caspase-3 and caspase-9. Moreover, it suppressed the activation of c-Jun-NH2-terminal kinase and p53 upregulated mediator of apoptosis, mechanisms closely associated with nonalcoholic steatohepatitis.^[147]^

### 4.5. Gout

In gout, xanthine oxidase (XO) orchestrates the oxidative hydroxylation pathway, converting hypoxanthine and subsequently into uric acid, precipitating the onset of excruciating inflammation.^[148]^ Nevertheless, *A vulgaris* exhibited a remarkable 95% inhibition of XO activity at a concentration of 100 µg/mL. Additionally, methanol extracts of *A vulgaris* exhibited robust XO-blocking effects, with IC50 values below 20 µg/mL.^[149,150]^ Furthermore, 50% ethanol extracts from *Artemisia selengensis* mitigated gout inflammation by inhibiting LPS-derived degradation of nuclear factor of kappa light polypeptide gene enhancer in B-cells inhibitor alpha, phosphorylation of p65, and upregulation of the nucleotide-binding oligomerization domain-like receptor containing pyrin domain 3 inflammatory complex. Furthermore, they suppressed the increased expression of active interleukin-1 beta and caspase-1 triggered by MSU and facilitated the movement of the nuclear factor E2-related factor 2 inside the nucleus, thus ameliorating the production of reactive oxygen species induced by MSU.^[151]^

## 5. Clinical evidence

Several studies have illustrated the efficacy of certain *Artemisia* species in diabetes management. Choi et al discovered that persistent daily intake of sajabalssuk ethanol extract (SBE) sourced from *A princeps* for 8 weeks notably diminished fasting blood glucose concentrations in hyperglycemic individuals. Furthermore, high-dose SBE demonstrated superior effectiveness compared to low-dose SBE in decreasing plasma levels of FFA and systolic blood pressure.^[152]^ Al-Waili et al discovered that, following treatment with *A herba-alba* aqueous extract, 14 of 15 patients with diabetes mellitus experienced significant alleviation of diabetic symptoms. Remarkably, no adverse effects were observed during or after administration.^[153]^ In addition, the study revealed that out of the 15 patients diagnosed with diabetes, *A herba-alba* aqueous extract was continued in 8 individuals. No adverse events were observed. Conversely, the extract was discontinued in the remaining 7 patients, who subsequently exhibited normal health during a follow-up period ranging from 3 to 8 months.^[154]^ An investigation revealed that among 119 people identified with type 2 diabetes undergoing treatment with SR2004 (containing *A dracunculus*), significant improvements were observed after 12 weeks. Hemoglobin A1c levels decreased from 9.0% to 7.1%, mean blood glucose dropped from 211 mg/dL to 133 mg/dL, mean TC level reduced to 185 mg/dL, and mean serum TG decreased to 160 mg/dL in 103 patients. Additionally, among the 13 patients who were on insulin at the beginning of the study, 5 patients only required oral hypoglycemics, while another 5 patients reduced their daily insulin dosage by 30%. Moreover, retinopathic changes were reversed in 2 patients.^[155]^ Li et al investigated 32 patients of both sexes, aged 30 to 60 years, diagnosed with type 2 diabetes. Dried powdered plant material of *Artemisia absinthium* was found to decrease HDL and LDL levels by 3% and 6%, respectively.^[156]^ Hassan et al observed a significant decrease in fasting serum glucose levels in the *A absinthium* dried powdered group.^[157]^ Mendez-del et al observed that providing ethanolic extract of *A dracunculus* for 90 days to individuals with impaired glucose tolerance resulted in a marked reduction in hemoglobin A1c, systolic blood pressure, and insulin bioavailability or area under the curve. Concurrently, there was a considerable increase in HDL-C levels.^[158]^ It was also found that *A scoparia* significantly lowered fasting plasma sugar levels, blood insulin concentration, HOMA-IR, and β-cell function. Furthermore, circulating adiponectin levels were notably increased. Regular intake of the extract can improve insulin sensitivity by increasing adiponectin levels in females with gestational diabetes.^[104]^

In an investigation involving patients with hyperlipidemia, it was found that supplementation of *A herba-alba* aqueous extract resulted in a notable decrease in blood cholesterol concentration.^[159]^ Furthermore, SPB-201 (powdered aqueous extract of *A annua*) led to a remarkable 271% reduction in aspartate aminotransferase levels, while a notable reduction of 334% in alanine aminotransferase levels was observed during the 4-week follow-up. This suggests an enhancement in liver function among subjects with mild-to-moderate NAFLD.^[160]^ Here, we reviewed the pharmacological effects of *Artemisia* on metabolic diseases. Pertinent clinical evidence is presented in Table 3.^[152–160]^

**Table 3:** Clinical evidence of *Artemisia* species for metabolic diseases.

Disease	Sample	Intervention measure (test/control group)	Dose	Duration	Outcome	References
T2DM	81 patients (men 52 and women 29), aged 25–65 yr	The low-dose SBE group/the high-dose SBE group/lactose/pinitol	1000 mg/570 mg/1000 mg/2000 mg, bid	8 wk	FBG↓, HbA1c↓, Plasma FFA↓, systolic blood pressure↓	[152]
T2DM	119 patients	SR2004	500 mg, tid	12 wk	blood glucose↓, HbA1c↓, TC↓, TG↓	[155]
T2DM	32 patients and aged 30–60 yr	*A absinthium*/*Gymnema sylvestre*/*Citrullus colocynthis*/placebo	500 mg, bid	30 d	HDL↓, LDL↓	[156]
T2DM	40 patients	*A absinthium*/*G sylvestre*/*C colocynthis*/placebo	1000 mg, qd	40 d	fasting serum glucose↓	[157]
IGT	24 patients	*A dracunculus*/placebo	1000 mg, bid	90 d	SBP↓, AUC↓, A1C↓, HDL-C↑	[158]
GDM	144 patients and aged 25–35 yr	*A scoparia* extract/placebo	400 mg, bid	10 wk	FBG↓, serum insulin levels↓, HOMA-IR↓, HOMA-B↓, APN↑	[104]
HLP	70 patients	*A. herba-alba* extract/simvastatin plus omega 3	100 mg, qd	60 d	cholesterol↓	[159]
NAFLD	170 patients	SPB-201/placebo	480 mg, bid	8 wk	AST↓, ALT↓	[160]

↑ indicates an increase, and ↓ indicates a decrease.

A1C = glycated hemoglobin, ALT = alanine aminotransferase, APN = adiponectin, AST = aspartate aminotransferase, AUC = area under the curve, FBG = fasting blood glucose, FFA = free fatty acid, GDM = gestational diabetes mellitus, HbA1c = hemoglobin A1c, HDL = high-density lipoprotein, HDL-C = high-density lipoprotein cholesterol, HLP = hyperlipidemia, HOMA-B = homeostasis model assessment of β-cell function, HOMA-IR = homeostasis model assessment of insulin resistance, IGT = impaired glucose tolerance, LDL = low-density lipoprotein, NAFLD = nonalcoholic fatty liver disease, SBE = sajabalssuk ethanol extract, SBP = systolic blood pressure, T2DM = type 2 diabetes mellitus, TC = total cholesterol, TG = triglyceride.

## 6. Conclusions and future perspective

### 6.1. Conclusions

*Artemisia,* a widely dispersed natural remedy, has been scrutinized for an extended period. As metabolic diseases emerge as a primary threat to human well-being, and with the inherent qualities of natural remedies offering minimal side effects, investigations into *Artemisia*’s efficacy in treating metabolic ailments have gained momentum. This review comprehensively outlines the chemical attributes, pharmacological effects, and molecular mechanisms associated with the therapeutic role of *Artemisia* in addressing metabolic disorders. This compilation of data serves as a resource for guiding future preclinical and clinical exploration of *Artemisia*. Both clinical and animal trials have attested to the efficacy of *Artemisia* species in managing metabolic diseases through various mechanisms. It has the potential to ameliorate diabetes by modulating blood glucose levels, enhancing insulin secretion, improving insulin sensitivity, and inhibiting oxidative stress and inflammation. Furthermore, in the treatment of diabetic nephropathy, *Artemisia* shows promise in ameliorating oxidative stress markers (such as SOD and MDA) and enhancing renal function by regulating key pathways (including toll-like receptor 4, S100A4, BCL-2-associated X, and B-cell lymphoma-2).^[161]^ Additionally, the modulation of nerve conduction velocity, neurotransmitter concentrations, coupled with the inhibition of 12/15-lipoxygenase and attenuation of nitrated protein expression, provides alleviation from diabetic neuropathy.^[162]^ In obesity management, various species of *Artemisia* have been shown to hinder adipogenesis, alleviate inflammation, and ameliorate gut microbiota dysbiosis. *Artemisia* contributes to the management of hyperlipidemia, NAFLD, and gout through diverse mechanisms. These include lowering blood lipids via AMPK activation, ameliorating hepatic steatosis and apoptosis through SREBP/AMPK/c-Jun-NH2-terminal kinase pathways, and inhibiting both XO and MSU-induced nucleotide-binding oligomerization domain-like receptor containing pyrin domain 3 inflammasome activation in gout.

The pharmacological effects of *Artemisia* species on metabolic disorders are attributed to their diverse phytochemical constituents. Among these, flavonoids, terpenoids, coumarins, and lignans have been identified as the major bioactive metabolites contributing to the therapeutic activities observed in the included studies.^[163]^ Flavonoids are the most extensively studied class of compounds in Artemisia species. Representative flavonoids such as apigenin and quercetin have been shown to improve insulin resistance by activating the AMPK and PI3K/Akt signaling pathways, reduce inflammation by inhibiting NF-κB activation and decreasing pro-inflammatory cytokines (e.g., TNF-α, IL-6),^[164–166]^ and alleviate oxidative stress by upregulating nuclear factor E2-related factor 2-mediated antioxidant enzymes (e.g., SOD, glutathione peroxidase).^[167,168]^ These multi-target effects make flavonoids key contributors to the hypoglycemic and hypolipidemic actions of *Artemisia* plants. Terpenoids, particularly artemisinin, have demonstrated anti-adipogenic effects. Evidence suggests that artemisinin can suppress adipogenesis by downregulating PPARγ and C/EBPα, thereby reducing lipid accumulation in adipose tissue and the liver.^[169]^ Coumarins such as scoparone have been reported to lower blood glucose levels by promoting insulin secretion from pancreatic β-cells and protecting against β-cell apoptosis, specifically exerting these effects by inhibiting cytokine-induced inducible NO synthase expression and NF-κB activation, thereby reducing NO production and preserving β-cell function.^[170]^ Lignans, including sesamin, reduce serum cholesterol, TG, blood glucose, and alanine transaminase, while increasing HDL-C. They also mitigate weight changes in diabetic models and lower body weight in metabolic syndrome models, due to their antioxidant and anti-inflammatory properties.^[171]^

### 6.2. Future perspectives

These findings underscore the efficacy of *Artemisia* spp. in combating metabolic disorders. However, certain shortcomings persist in current research. While this review catalogs numerous species of *Artemisia* known to alleviate metabolic disorders, further investigation is imperative to ascertain whether all *Artemisia* species can effectively manage such conditions. Despite the identification of many compounds in this species, definitive determination of the primary active ingredients for metabolic disorder treatment remains elusive. Second, there is limited research on the effects of *Artemisia* on complications associated with diabetes, particularly concerning the chronic damage caused by elevated blood glucose levels, which imperil major blood vessels, microvessels, and vital organs, such as the brain, heart, and kidneys. Notably, the safety profile of *Artemisia* in clinical therapeutics is of paramount importance. Administering high doses exceeding 3 g/kg of *Artemisia* for a certain period may elicit adverse effects, such as accelerated respiration, neurotoxicity, and reproductive toxicity.^[14]^ These findings underscore the importance of caution in the clinical use of *Artemisia*. Furthermore, to elucidate the mechanism of action of *Artemisia* in the human body and ascertain its optimal dosage, treatment duration, and potential adverse effects, it is imperative to conduct further clinical investigations on *Artemisia.* In summary, *Artemisia* spp. is a promising therapeutic agent for treating metabolic disorders. Hence, elucidating its chemical composition, unraveling its molecular mechanisms and target actions, and evaluating its clinical efficacy and safety are essential steps toward advancing its development and clinical utilization.

## Acknowledgments

We are thankful to the Qilu Institute of Technology for their contribution to this work. We are grateful to the National Science Foundation of Shandong Province, China, for funding this work.

## Author contributions

**Conceptualization:** Naiyu Wang, Zheng Wang, Hualin Wang, Hailing Ding, Muhammad Shahbaz, Muhammad Ijaz.

**Data curation:** Minmin Li, Muhammad Shahbaz, Muhammad Ijaz.

**Formal analysis:** Minmin Li, Zheng Wang.

**Methodology:** Mingjing Lu, Hualin Wang, Meiyue Yao.

**Funding acquisition:** Hailing Ding.

**Resources:** Mingjing Lu, Muhammad Ijaz.

**Software:** Mingjing Lu.

**Investigation:** Zheng Wang, Hualin Wang.

**Project administration:** Meiyue Yao, Hailing Ding.

**Writing – original draft:** Naiyu Wang, Muhammad Ijaz.

**Writing – review & editing:** Naiyu Wang, Muhammad Shahbaz, Muhammad Ijaz.
